# Dataset of Grey plover (*Pluvialissquatarola*) diet composition on the SW Black Sea coast using DNA metabarcoding

**DOI:** 10.3897/BDJ.13.e150802

**Published:** 2025-07-07

**Authors:** Liliana V. Vassileva, Lyudmila Lozanova, Martin Marinov, Jérôme Morinière, Boyko Neov, Boris Nikolov, Nikolay Simov, Stefania Klayn

**Affiliations:** 1 Institute of Biodiversity and Ecosystem Research, Bulgarian Academy of Sciences, Sofia, Bulgaria Institute of Biodiversity and Ecosystem Research, Bulgarian Academy of Sciences Sofia Bulgaria; 2 AIM - Advanced Identification Methods GmbH, Leipzig, Germany AIM - Advanced Identification Methods GmbH Leipzig Germany; 3 National Museum of Natural History, Bulgarian Academy of Sciences, Sofia, Bulgaria National Museum of Natural History, Bulgarian Academy of Sciences Sofia Bulgaria

**Keywords:** shorebirds, Black-bellied plover, High Throughput Sequencing (HTS), Illumina MiSeq, coastal food webs, south-western Black Sea, environmental DNA, CO1

## Abstract

**Background:**

Wader populations have been declining globally due to widespread anthropogenic habitat degradation and loss. In this context, food quality and availability at migration stop-over sites is crucial for wader migration success.

We studied the diet composition and preferences of the Grey plover (*Pluvialissquatarola*) at a stop-over site on the Black Sea-Mediterranean Flyway - Pomorie Lake, SW Black Sea coast, Bulgaria - through DNA metabarcoding of faeces. The study aimed to determine the bird's feeding strategy, coastal habitat use and possible seasonal variations as a result of local prey availability.

**New information:**

The dataset includes new data on Grey plover (*Pluvialissquatarola*) prey items on the south-western Black Sea coast, Bulgaria. The diet composition data provides valuable insights on coastal biodiversity in the area as "sampled" by this generalist invertivore and contributes to the knowledge of Grey plover prey choice and habitat use in a little-studied microtidal marine region.

## Introduction

Long-distance migration between wintering and breeding grounds is one of the most energetically demanding processes in the animal kingdom and is well known in many species of waders. Most migratory birds need to make several stop-overs to rest and refuel for the next stages of their migration (Schaub and Jenni 2000, Skagen 2006). High-quality stop-over sites, with sufficient resources and protection from disturbance, are essential for bird survival and migration success (Myers et al. 1987, McGowan et al. 2011). Coastal and inland wetlands are often preferred stop-over sites; however, in recent years, these habitats have been subjected to fragmentation, changes in hydrology and land use, organic and/or chemical pollution etc. due to human activities and climate change, leading to their deterioration at a global scale (Finlayson et al. 2018).

The Grey plover (*Pluvialissquatarola*) is a long-distance migratory wader which breeds in the Arctic and uses stop-overs at tropical, subtropical and temperate mudflats, bea­ches and coasts worldwide (Byrkjedal and Thompson 1998). On the Black Sea coast (part of the Black Sea-Mediterranean Flyway (BSMF)), it can be observed in spring, autumn and winter (Nankinov 1996, Michev and Profirov 2003). The species is protected under the Bulgarian Biodiversity Act and its populations are declining globally.

The Grey plover is a generalist feeder with a broad diet spectrum. In western Europe, its most common prey during the non-breeding season include polychaetes, small bivalves and crustaceans, with diet variations depending on location, habitat, season and sub­strate (Pienkowski 1982, Bocher et al. 2014, Poole et al. 2020). The Grey plover’s diet on the BSMF is poorly studied (Prostov 1964, Chernichko 2015, Kirikova 2017, Khrokov 2019).

Wader diet is typically analysed by morphological taxonomic identification of the prey remains in stomach contents or faeces (Verkuil 1996) - a time-consuming process that requires specific taxonomic expertise. Many invertebrate prey species decompose quickly and/or lack hard indigestible parts; therefore, they tend to remain undetected or underestimated (Perez-Hurtado et al. 1997). Considering the generalist feeding strategy and the wide diet spectrum reported for Grey plover along its migratory routes, as well as the lack of data for the Black Sea coast, we used DNA metabarcoding of faecal samples to determine the bird’s diet composition in the Black Sea coastal habitats. Environmental DNA (eDNA) is a non-invasive method of diet study (Pompanon et al. 2012) that is increasingly applied in ecological research (e.g. Stolz et al. (2023), Zurdo et al. (2023)). Provided that the reference molecular databases for the target species are sufficiently populated with sequences from reliably identified specimens (Meiklejohn et al. 2019, Pentinsaari et al. 2020, Keck et al. 2023), the method can give unprecedented taxonomic resolution in prey identification and allow the detection of cryptic or decom­posed prey and even of unexpected prey items (e.g. Gerwing et al. (2016), Novcic et al. (2016), McInnes et al. (2017), Heller (2020)). de Sousa et al. (2019) even introduced the term dietary DNA (dDNA) to complement eDNA for dietary purposes and local biodiversity description.

This paper presents the raw CO1 dataset of obtained sequences published and available in BOLD. The results are discussed and analysed in detail in Vassileva et al. (2024).

## Sampling methods

### Sampling description

Seventy-six Grey plover faecal samples were collected at Pomorie Lake (SW Black Sea coast) in October–November 2020 (n = 61) and in March 2021 (n = 15), preserved in 95% ethanol and sent to AIM - Advanced Identification Methods GmbH, Germany for DNA extraction and metabarcoding. Samples were collected immediately after bird defecation was observed in situ; to further ensure that they indeed belonged to Grey plovers, no attempt was made to block host DNA during amplification.

Molecular analyses and bioinformatics are described in detail in Vassileva et al. (2024).

Briefly, species identification of faecal organic material was performed using DNA metabarcoding following the protocol of Hausmann et al. (2020). Initially, two molecular markers were targeted: CO1 for animal and ITS2 - for plant identification. Subsequently, only CO1 results were retained for the analyses, since many fewer taxa with a low number of reads were detected by ITS2 in only a few samples, suggesting that the birds targeted animal prey exclusively. The current dataset includes only the CO1 sequences, but the ITS2 sequences have also been published and are available in GenBank (accession numbers PP060518-PP060554) . For amplification of the CO1 target region, Illumina-ready primers derived from the primer pair by Leray et al. (2013) (dgHCO 5′-GGWACWGGWTGAACWGTWTAYCCYCC-3’ mlCOIntF 5′-TAAACTTCAGGGTGACCAAARAAYCA-3′) were used, with forward and reverse HTS primers equipped with complementary sites for the Illumina sequencing tails. High-throughput sequencing (HTS) was performed on an Illumina MiSeq. No attempt was made to block the amplification of the host DNA during PCR in order to confirm that the collected faeces indeed belonged to Grey plovers.

The obtained sequences were clustered into operational taxonomic units (OTUs), which were blasted against: (1) a custom database downloaded from GenBank (a local copy of the NCBI nucleotide database downloaded from ftp://ftp.ncbi.nlm.nih.gov/blast/db/) and (2) a custom database created from data downloaded from BOLD (www.boldsystems.org), including taxonomy and BIN information, using Geneious (v.10.2.5 - Biomatters, Auckland, New Zealand) and following the methods described in Morinière et al. (2016). As an additional control measure, the OTUs were classified into taxa using the naive Bayesian classifier of the Ribosomal Database Project (RDP), trained on a curated CO1 dataset of arthropods and chordates (plus outgroups; see Porter and Hajibabaei (2018)). Further details on the bioinformatics procedures can be found in the supplements of Uhler et al. (2021).

## Geographic coverage

### Description

The faecal samples were collected on the sand stripe between Pomorie Lake (part of the Burgas Lakes complex, south-western Black Sea coast, Bulgaria) and the Black Sea. The study area is located on the Black Sea - Mediterranean Flyway.

### Coordinates

 and 42.62154 Latitude; and 27.63367 Longitude.

## Taxonomic coverage

### Taxa included

**Table:** 

Rank	Scientific Name	
phylum	Annelida	
phylum	Arthropoda	
phylum	Ascomycota	
phylum	Bacillariophyta	
phylum	Basidiomycota	
phylum	Chlorophyta	
phylum	Choanozoa	
phylum	Chordata	
phylum	Cnidaria	
phylum	Eukarya_unassigned	
phylum	Evosea	
phylum	Mollusca	
phylum	Nematoda	
phylum	Ochrophyta	
phylum	Oomycota	
phylum	Platyhelminthes	
phylum	Porifera	
phylum	Rhodophyta	
phylum	Tardigrada	
class	Clitellata	
class	Polychaeta	
class	Arachnida	
class	Branchiopoda	
class	Chilopoda	
class	Collembola	
class	Diplopoda	
class	Hexanauplia	
class	Insecta	
class	Malacostraca	
class	Dothideomycetes	
class	Eurotiomycetes	
class	Lecanoromycetes	
class	Leotiomycetes	
class	Saccharomycetes	
class	Sordariomycetes	
class	Bacillariophyceae	
class	Coscinodiscophyceae	
class	Agaricomycetes	
class	Mamiellophyceae	
class	Choanoflagellatea	
class	Actinopterygii	
class	Aves	
class	Mammalia	
class	Hydrozoa	
class	Eumycetozoa	
class	Bivalvia	
class	Gastropoda	
class	Chromadorea	
class	Phaeophyceae	
class	Monogenea	
class	Trematoda	
class	Demospongiae	
class	Florideophyceae	
class	Heterotardigrada	
order	Haplotaxida	
order	Opisthopora	
order	Phyllodocida	
order	Sabellida	
order	Spionida	
order	Araneae	
order	Ixodida	
order	Opiliones	
order	Sarcoptiformes	
order	Anomopoda	
order	Lithobiomorpha	
order	Entomobryomorpha	
order	Julida	
order	Cyclopoida	
order	Blattodea	
order	Coleoptera	
order	Dermaptera	
order	Diptera	
order	Ephemeroptera	
order	Hemiptera	
order	Hymenoptera	
order	Lepidoptera	
order	Neuroptera	
order	Orthoptera	
order	Psocodea	
order	Thysanoptera	
order	Trichoptera	
order	Amphipoda	
order	Decapoda	
order	Isopoda	
order	Capnodiales	
order	Cladosporiales	
order	Eurotiales	
order	Lecanorales	
order	Saccharomycetales	
order	Glomerellales	
order	Bacillariales	
order	Naviculales	
order	Thalassiosirales	
order	Agaricales	
order	Mamiellales	
order	Craspedida	
order	Blenniiformes	
order	Charadriiformes	
order	Carnivora	
order	Primates	
order	Leptothecata	
order	Siphonophorae	
order	Physariida	
order	Mytilida	
order	Venerida	
order	Littorinimorpha	
order	Rhabditida	
order	Fucales	
order	Peronosporales	
order	Plagiorchiida	
order	Axinellida	
order	Ceramiales	
order	Corallinales	
order	Echiniscoidea	
family	Lumbricidae	
family	Nereididae	
family	Polynoidae	
family	Sabellariidae	
family	Spionidae	
family	Araneidae	
family	Lycosidae	
family	Tetragnathidae	
family	Ixodidae	
family	Phalangiidae	
family	Lithobiidae	
family	Tomoceridae	
family	Julidae	
family	Ectobiidae	
family	Cantharidae	
family	Carabidae	
family	Cerambycidae	
family	Chrysomelidae	
family	Cleridae	
family	Coccinellidae	
family	Curculionidae	
family	Elateridae	
family	Melyridae	
family	Staphylinidae	
family	Tenebrionidae	
family	Forficulidae	
family	Agromyzidae	
family	Anthomyiidae	
family	Asilidae	
family	Bibionidae	
family	Calliphoridae	
family	Cecidomyiidae	
family	Ceratopogonidae	
family	Chironomidae	
family	Chloropidae	
family	Conopidae	
family	Dolichopodidae	
family	Drosophilidae	
family	Ephydridae	
family	Fanniidae	
family	Heleomyzidae	
family	Hybotidae	
family	Limoniidae	
family	Lonchaeidae	
family	Lonchopteridae	
family	Milichiidae	
family	Muscidae	
family	Mycetophilidae	
family	Phoridae	
family	Polleniidae	
family	Psychodidae	
family	Rhagionidae	
family	Rhiniidae	
family	Sarcophagidae	
family	Scathophagidae	
family	Sciaridae	
family	Simuliidae	
family	Sphaeroceridae	
family	Syrphidae	
family	Tachinidae	
family	Tephritidae	
family	Tipulidae	
family	Baetidae	
family	Acanthosomatidae	
family	Aphididae	
family	Aphrophoridae	
family	Cicadellidae	
family	Coreidae	
family	Issidae	
family	Pentatomidae	
family	Psyllidae	
family	Rhyparochromidae	
family	Andrenidae	
family	Apidae	
family	Braconidae	
family	Colletidae	
family	Crabronidae	
family	Diapriidae	
family	Eulophidae	
family	Formicidae	
family	Halictidae	
family	Ichneumonidae	
family	Megachilidae	
family	Perilampidae	
family	Pompilidae	
family	Pteromalidae	
family	Tenthredinidae	
family	Vespidae	
family	Blastobasidae	
family	Crambidae	
family	Erebidae	
family	Geometridae	
family	Hepialidae	
family	Incurvariidae	
family	Lypusidae	
family	Noctuidae	
family	Nymphalidae	
family	Oecophoridae	
family	Pieridae	
family	Pyralidae	
family	Tortricidae	
family	Mantispidae	
family	Acrididae	
family	Tettigoniidae	
family	Psocidae	
family	Stenopsocidae	
family	Phlaeothripidae	
family	Hydropsychidae	
family	Alpheidae	
family	Grapsidae	
family	Polybiidae	
family	Ischnomesidae	
family	Cladosporiaceae	
family	Aspergillaceae	
family	Trichocomaceae	
family	Ramalinaceae	
family	Saccharomycetaceae	
family	Bacillariaceae	
family	Pinnulariaceae	
family	Skeletonemataceae	
family	Mamiellaceae	
family	Salpingoecidae	
family	Blenniidae	
family	Charadriidae	
family	Laridae	
family	Canidae	
family	Hominidae	
family	Apolemiidae	
family	Prayidae	
family	Mytilidae	
family	Mesodesmatidae	
family	Littorinidae	
family	Rhabditidae	
family	Sargassaceae	
family	Peronosporaceae	
family	Callithamniaceae	
family	Rhodomelaceae	
family	Echiniscoididae	

## Temporal coverage

**Data range:** 2020-10-25 – 2021-3-27.

### Notes

In the BOLD dataset, all sequences were assigned the date 2020-10-31 for simplicity, as they were usually present in multiple samples from different dates, but required a single entry in the database. This is reflected in the collection notes (fieldNotes).

## Usage licence

### Usage licence

Open Data Commons Attribution License

## Data resources

### Data package title

*Pluvialissquatarola* faeces metabarcoding - CO1

### Resource link

http://doi.org/10.5883/DS-PLUVS

### Number of data sets

1

### Data set 1.

#### Data set name

*Pluvialissquatarola* faeces metabarcoding - CO1 [BOLD_DATASET_CODES:DS-PLUVS]

#### Data format

Darwin Core

#### Description

The dataset contains 429 records (DNA sequences identified with CO1 from *P.squatarola* faeces), of which 244 records with BOLD Barcode Index Numbers (BINs) and 158 - with species assignment.

**Figure 1.**
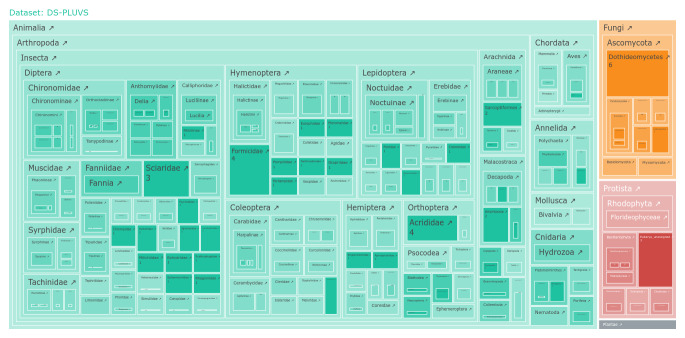
Treemap of the taxonomic composition of sequences identified in the *P.squatarola* faecal samples. An interactive version of the plot can be found on the dataset's page in BOLD.

Arthropoda is the most represented group, constituting 78% of the records in the dataset. A total of 91% of all arthropod records (or 71% of all records) belong to Insecta , with Diptera, Hymenoptera and Lepidoptera the most represented groups. The other taxonomic groups have relatively minor shares (Fig. 1).

The dataset also includes metadata on the field methods (sampling method, geographic location, date), as well as the taxonomy (taxonomic identification methods, taxonomic classification) and the exact nucleotide sequence of each record.

**Data set 1.** 

Column label	Column description
materialSampleID	BOLD process ID - unique identifier for each record in BOLD.
recordNumber	BOLD sample ID - unique identifier for each record (sequence).
fieldNumber	BOLD field ID - unique field/sample identifier for each record (sequence), here identical to recordNumber.
occurrenceID	BOLD specimen ID - unique occurrence identifier for each record (sequence).
measurementMethod	BOLD Barcode Index Number (BIN) uniform resource identifier (URI) corresponding to the record (sequence).
institutionCode	Name of the institution having custody of the sequences.
kingdom	Full scientific name of the kingdom of the taxon to which the sequence is assigned.
phylum	Full scientific name of the phylum or division of the taxon to which the sequence is assigned.
class	Full scientific name of the class of the taxon to which the sequence is assigned.
order	Full scientific name of the order of the taxon to which the sequence is assigned.
family	Full scientific name of the family of the taxon to which the sequence is assigned.
subfamily	Full scientific name of the subfamily of the taxon to which the sequence is assigned.
tribe	Full scientific name of the tribe of the taxon to which the sequence is assigned.
genus	Full scientific name of the genus of the taxon to which the sequence is assigned.
scientificName	Full scientific name of the taxon to which the sequence is assigned.
scientificNameAuthorship	Authorship information for the scientific name (field scientificName) of the taxon to which the sequence is assigned.
identificationRemarks	Method used to assign a taxonomic identity to the sequence.
identifiedBy	Name of the person/people who assigned the taxon identification to the sequence.
typeStatus	Status of the specimen voucher in BOLD.
materialSample	Type of tissue/material sampled from which the DNA sequences were derived.
recordedBy	Name of the person/people who collected the original field samples.
eventDate	Field sample collection date.
fieldNotes	Additional notes related to the field sampling.
country/waterBody	Country in which the field sampling occurred.
countryCode	Standard two-letter code for the country in which the field sampling occurred.
stateProvince	Full name of the NUTS3 (Nomenclature of territorial units for statistics level 3) administrative region in which the field sampling occurred.
county	Full name of the municipality in which the field sampling occurred.
municipality	Full name of the Natura 2000 area in which the field sampling occurred.
verbatimLocality	Additional text description of the exact location of the collection site.
decimalLatitude & decimalLongitude	Geographic latitude and longitude of the sampling location in decimal degrees (WGS84, EPSG 4326).
coordinatePrecision	Coordinate precision of the field decimalLatitude & decimalLongitude.
georeferenceSources	Resources used to georeference the sampling location.
verbatimElevation	Original description of the elevation (altitude above sea level in metres) of the sampling location.
samplingProtocol	Sampling protocol used for the field sampling.
dynamicProperties	Exact nucleotide sequence corresponding to the record.
measurementDeterminedBy	Institution that uploaded and curated the dataset.
measurementDeterminedDate	Date of upload of the dataset to BOLD.
datasetName	Dataset unique identifier in BOLD.

## Additional information

Some of the sequences are likely contamination (e.g. most of Chordata, soil fungi) and others are ecto- and endoparasites (e.g. Ixodida, Platyhelminthes, Nematoda). While these cannot be considered part of the Grey plover's diet and were, therefore, excluded from the published diet analyses (Vassileva et al. 2024), they are included in this dataset for the sake of completeness and biodiversity listing purposes. That said, a critical review and conservative ecological approaches should be applied to the raw metabarcoding data prior to their validation. A thorough verification against the local ecological communities is highly recommended in order to attribute them to the living systems.
